# Risk factors and geographic disparities in premature cardiovascular mortality in US counties: a machine learning approach

**DOI:** 10.1038/s41598-023-30188-9

**Published:** 2023-02-20

**Authors:** Weichuan Dong, Issam Motairek, Khurram Nasir, Zhuo Chen, Uriel Kim, Yassin Khalifa, Darcy Freedman, Stephanie Griggs, Sanjay Rajagopalan, Sadeer G. Al-Kindi

**Affiliations:** 1 Department of Population and Quantitative Health Sciences, Case Western Reserve University School of Medicine, Cleveland, OH 44106 USA; 2 Harrington Heart and Vascular Institute, University Hospitals, 11100 Euclid Ave, Cleveland, OH 44106 USA; 3 Houston Methodist Hospital, Houston, TX 77030 USA; 4 Kellogg School of Management, Northwestern University, Evanston, IL 60208 USA; 5 Mary Ann Swetland Center for Environmental Health, Case Western Reserve University, Cleveland, OH 44106 USA; 6 Frances Bolton School of Nursing, Case Western Reserve University, Cleveland, OH 44106 USA; 7 Case Western Reserve University School of Medicine, Cleveland, OH 44106 USA

**Keywords:** Epidemiology, Cardiology, Risk factors, Health care, Disease prevention, Health services, Public health, Cardiovascular diseases

## Abstract

Disparities in premature cardiovascular mortality (PCVM) have been associated with socioeconomic, behavioral, and environmental risk factors. Understanding the “phenotypes”, or combinations of characteristics associated with the highest risk of PCVM, and the geographic distributions of these phenotypes is critical to targeting PCVM interventions. This study applied the classification and regression tree (CART) to identify county phenotypes of PCVM and geographic information systems to examine the distributions of identified phenotypes. Random forest analysis was applied to evaluate the relative importance of risk factors associated with PCVM. The CART analysis identified seven county phenotypes of PCVM, where high-risk phenotypes were characterized by having greater percentages of people with lower income, higher physical inactivity, and higher food insecurity. These high-risk phenotypes were mostly concentrated in the Black Belt of the American South and the Appalachian region. The random forest analysis identified additional important risk factors associated with PCVM, including broadband access, smoking, receipt of Supplemental Nutrition Assistance Program benefits, and educational attainment. Our study demonstrates the use of machine learning approaches in characterizing community-level phenotypes of PCVM. Interventions to reduce PCVM should be tailored according to these phenotypes in corresponding geographic areas.

## Introduction

Premature cardiovascular mortality (PCVM) remains the leading cause of death in people under 65 years of age in the United States^1^. Although cardiovascular mortality rates in the overall population have declined steadily over the past decades, recent evidence shows that improvements in younger individuals have plateaued^2,3^. Additional evidence suggests that the burden of cardiovascular disease (CVD) mortality is unevenly distributed across geographic areas^4^. There is a substantial geographical variation in CVD mortality across US counties, most pronounced for ischemic heart disease and cerebrovascular disease^4^. Similar trends have also been observed for PCVM^5^.

Previous studies have linked socioeconomic, behavioral, and environmental risk factors to PCVM outcomes using composite metrics and indices, such as the social vulnerability and the sociodemographic indices^6,7^. However, to our knowledge, no prior study has investigated the community-level phenotypes of PCVM, or the combinations of risk factors highly associated with PCVM, as well as their geographic distributions. In this study, we identified county phenotypes of PCVM in the US from a broad range of risk factors using novel machine learning approaches. By presenting these phenotypes on a map, we were able to locate areas of high PCVM risk and their associated characteristics. We additionally compared the relative importance of risk factors in predicting PCVM.

Identifying not only the clusters of high PCVM but also the clusters of PCVM phenotypes acknowledges the complex relationships among selected drivers of high PCVM and PCVM disparities. This place-specific phenotype approach offers researchers and practitioners a framework for addressing community-level disparities in PCVM.

## Methods

### Study population

Our study population included individuals aged 15–64 years who died from CVD during the years 2015–2019 in the contiguous United States. PCVM was defined as the number of deaths in persons aged 15–64 years caused by CVD per 100,000 people at the county level, age-adjusted to the 2000 US Standard Population. We only included counties with at least 20 deaths from CVD during the study period to mitigate against unstable PCVM estimates. Counties from Hawaii and Alaska were excluded from the analysis due to the lack of complete risk factor data.

### Data sources

Mortality data were accessed through the multiple cause of death files, maintained by the National Center for Health Statistics via the Centers for Disease Control and Prevention Wide-ranging Online Data for Epidemiologic Research (CDC-WONDER) database^8^. This database contains death certificates data from all fifty states, with cause of death identified by the international classification of disease, version 10 (ICD-10) coding schema. Data from the CDC-WONDER also include age at death, sex, race, and county of death. If multiple underlying causes of death on the death certificate are noted, a single cause is inserted according to the sequence of conditions on the certificate and contributing causes of death according to prespecified methods^8^. ICD-10 codes for CVD mortality were defined as follows: ischemic heart disease (I20–I25), heart failure (I50), cerebrovascular diseases (I60–I69), and hypertensive heart disease (I10–I15).

County-level risk factor data were harvested from a variety of data sources (Table 1), including County Health Rankings & Roadmaps^9^, Area Health Resources Files^10^, and Environmental Protection Agency’s Environmental Justice Screening tool (EJSCREEN)^11^. To best align temporally with the PCVM data, we used risk factor data collected in 2017 (the mid-year of the PCVM data) or the year closest to 2017. We used the 2020 EJSCREEN data (covering the years 2014–2020), and we re-estimated county-level exposures using the method outlined by the EPA EJSCREEN technical documentation guide since EJSCREEN data is natively reported at the census block group level^11^. We also visualized the geographic distributions of all county-level risk factors used in the study (Supplemental Fig. S1).

**Table 1:** Definition and summary statistics of risk factors in the study.

Variables	Description	Year	Original source	Mean (SD)
Race/ethnicity				
Hispanic	Percentage of population identifying as Hispanics	2017	Census—PE	9.2 (13.3)
Non-hispanic black	Percentage of Non-Hispanic African American population	2017	Census—PE	10.6 (15.1)
Non-hispanic white	Percentage of Non-Hispanic White population	2017	Census—PE	75.4 (19.8)
Asian and Pacific Islander	Percentage of Asian and Pacific Islander population	2017	Census—PE	1.7 (2.7)
Population structure				
Population age ≤ 18	Percentage of population age 18 years or younger	2017	Census—PE	22.2 (3.2)
Population age 65 +	Percentage of population age 65 years or older	2017	Census—PE	18.2 (4.2)
Population female	Percentage of female population	2017	Census—PE	50.2 (2.0)
Rural population	Percentage of people living in rural areas	2010	Census—PE	52.4 (29.5)
Environmental exposure				
PM2.5 level in air	PM_2.5_ levels in air, µg/m^3^ (annual average)	2018	EPA	8.0 (1.2)
Air toxics respiratory hazard index	Ratio of exposure concentration to health-based reference concentration	2017	EPA	0.39 (0.14)
Ozone level in air	Ozone summer seasonal average of daily maximum 8 h concentration in air in parts per billion	2018	EPA	41.5 (4.9)
Diesel PM level in air	Diesel particulate matter level in air, µg/m^3^	2017	EPA	0.24 (0.16)
Traffic proximity and volume	Average annual daily count of vehicles at major roads within 500 m, divided by distance in meters	2019	EPA	181.2 (318.1)
Pre-1960 housing	Fraction of housing units built pre-1960, as indicator of potential lead paint exposure	2016–2020	Census—ACS	0.27 (0.14)
Proximity to RMP sites	Count of RMP (potential chemical accident management plan) facilities within 5 km (or nearest one beyond 5 km), each divided by distance in km	2020	EPA	0.49 (0.48)
Proximity to hazardous waste facilities	Count of hazardous waste facilities within 5 km (or nearest beyond 5 km), each divided by distance in km	2020	EPA	0.84 (5.23)
Proximity to NPL sites	Count of proposed or listed NPL—also known as superfund—sites within 5 km (or nearest one beyond 5 km), each divided by distance in km	2020	EPA	0.07 (0.10)
Major dischargers to water indicator	Modeled Toxic Concentrations at stream segments within 500 m, divided by distance in km	2019	EPA	16.2 (622.2)
Socioeconomic status				
Income inequality	Ratio of household income at the 80th percentile to income at the 20th percentile	2015–2019	Census—ACS	4.6 (0.7)
High school degree	Percentage of people (aged ≥ 25 year) and over with a high school diploma or equivalent	2015–2019	Census—ACS	86.5 (5.9)
College degree	Percentage of people (aged 25–44 year) with some post-secondary education	2015–2019	Census—ACS	57.5 (11.4)
Unemployment	Percentage of people (aged ≥ 16 year) unemployed but seeking work	2017	BLS	4.8 (1.5)
Median Household Income	The income (US dollar) where half of households in a county earn more and half of households earn less	2017	AHRF	51,231 (14,030)
Poverty	Percentage of people whose income under the federal poverty level	2017	AHRF	15.8 (6.3)
Under 200% poverty	Percentage of people (aged 18–64 year) whose income is under 200% of the federal poverty level	2017	AHRF	33.3 (9.2)
Receipt of SNAP benefits	Percentage of people who were food stamp recipients	2017	AHRF	14.3 (6.8)
Not proficient in English	Percentage of people (aged ≥ 5 year) who reported speaking English less than very well	2015–2019	Census—ACS	1.7 (2.6)
Severe housing problems	Percentage of households with at least 1 of 4 housing problems: overcrowding, high housing costs, lack of kitchen facilities, or lack of plumbing facilities	2013–2017	CHAS	14.0 (3.8)
Severe housing cost burden	Percentage of households that spend 50% or more of their household income on housing	2015–2019	Census—ACS	11.4 (3.3)
Homeownership	Percentage of owner-occupied housing units	2015–2019	Census—ACS	71.1 (8.2)
Broadband access	Percentage of households with broadband internet connection	2015–2019	Census—ACS	75.7 (8.9)
Social associations	Number of membership associations per 10,000 population	2017	CBP	11.2 (4.3)
Health status				
Diabetes	Percentage of adults (age ≥ 20) with diagnosed diabetes (age-adjusted)	2017	CDC—DSS	12.7 (3.7)
Low birthweight	Percentage of live births with low birthweight (< 2500 g)	2013–2019	CDC—NCHS	8.4 (1.9)
Sexually transmitted infections	Number of newly diagnosed chlamydia cases per 100,000 people	2017	CDC—NCHHSTP	419.1 (246.5)
Health behavior				
Adult obesity	Percentage of the adult population (aged ≥ 18 year) that reports a body mass index ≥ 30 (age-adjusted)	2017	CDC—DSS	34.0 (5.8)
Insufficient sleep	Percentage of adults who report fewer than 7 h of sleep on average (age-adjusted)	2018	CDC—BRFSS	37.5 (3.8)
Excessive drinking	Percentage of adults reporting binge or heavy drinking (age-adjusted)	2017	CDC—BRFSS	17.4 (3.2)
Adult smoking	Percentage of adults who are current smokers (age-adjusted)	2017	CDC—BRFSS	17.9 (3.5)
Physical inactivity	Percentage of adults (age ≥ 18 year) reporting no leisure-time physical activity (age-adjusted)	2017	CDC—DSS	27.1 (6.0)
Flu vaccinations	Percentage of fee-for-service Medicare enrollees that had an annual flu vaccination	2017	CMS—MMD	43.5 (8.5)
Food insecurity	Percentage of people who lack adequate access to food	2017	MMG	13.6 (4.0)
Limited access to healthy foods	Percentage of people who are low-income and do not live close to a grocery store	2015	USDA—FEA	7.2 (5.5)
Access to exercise opportunities	Percentage of people with adequate access to locations for physical activity	2010 & 2019	ESRI & Census—TF	64.9 (21.8)
Driving alone to work	Percentage of the workforce that drives alone to work (indicators of physical inactivity and the transit system)	2015–2019	Census—ACS	81.1 (5.8)
Long commute-driving alone	Among workers who commute in their car alone, the percentage that commute more than 30 min (indicator of physical inactivity)	2015–2019	Census—ACS	33.5 (12.1)
Clinical care				
Uninsured rate	Percentage of people (aged 18–64 year) without health insurance	2017	HRSA—AHRF	13.2 (6.0)
Primary care physicians	Primary care physicians in patient care per 100,000 people	2017	HRSA—AHRF	53.7 (33.0)
Hospitals	Hospitals per 100,000 people	2019	HRSA—AHRF	3.5 (3.5)
Community health centers	Community health centers per 100,000 people	2017	HRSA—AHRF	5.5 (9.1)

Variables obtained from AHRF: all variables under Clinical Care, Receipt of SNAP benefits, Median Household Income, Poverty, Under 200% poverty; Variables obtained from EPA-EJSCREEN: all variables under Environmental Exposure; Variables obtained from CHR: all other variables.

*AHRF* area health resources files, *BLS* bureau of labor statistics, *BRFSS* behavioral risk factor surveillance system, *CBP* county business patterns, *CDC* centers for disease control and prevention, *CHAS* comprehensive housing affordability strategy, *CHF* county health rankings & roadmaps, *CMS* centers for medicare & medicaid services, *DSS* US diabetes surveillance system, *EJSCREEN* environmental justice screening tool, *EPA* environmental protection agency, *FEA* food environment atlas, *HRSA* health resources and services administration, *MMD* mapping medicare disparities (MMD) tool, *MMG* map the meal gap, *NCHHSTP* national center for HIV/AIDS, viral hepatitis, STD, and TB prevention, *NCHS* national center for health statistics, *NLP* national priorities list, *PE* population estimates, *PM* fine particulate matter, *RMP* risk management plan, *SNAP* supplemental nutrition assistance program, *TF* tigerline files, *USDA* US department of agriculture.

Given the deidentified nature of the data and no individual-level data was used, institutional review board approval was not required.

### Statistical analysis

We applied CART and random forest machine learning methods and geographic information systems to explore the association between county-level risk factors and PCVM. CART was used to identify phenotypes of PCVM, or combinations of county-level characteristics that were associated with PCVM^12^. We performed additional analyses to examine whether the county-level mortality rates for each subtype of PCVM (i.e., heart failure, hypertension, ischemic heart disease, and stroke) have a similar pattern after group them according to the phenotypes identified by the main model. Finally, we used random forest analysis^13^ to examine the relative importance of risk factors in predicting PCVM. We compared the concordance between the CART and random forest models, with a key focus on whether high-importance variables from the random forest models were included in the phenotypes identified by the CART analysis.

CART uses conditional inference to recursively partition data into smaller and homogeneous groups characterized by combinations of predictors^14,15^. At each split, the data are divided into two groups by an algorithm-selected variable and a threshold value that maximizes the difference between the split groups. The splitting procedure recursively repeats for each split group until some user-defined stopping criteria are met. We set the following stopping criteria: a maximum tree depth of six splits, a minimum number of 200 counties in a terminal node, and a statistical significance for variable splits (α < 0.05) using the Pearson correlation test. Each terminal node of the tree consists of a group of counties with similar levels of PCVM. The combination of characteristics associated with a terminal node represents a phenotype of PCVM. We then used geographic information systems to visualize the distribution of the identified phenotypes.

The CART models were established using a randomly sampled training set (consisting of 80% of all counties) and the results were validated against the test set (consisting of the rest 20% of the counties). To validate the results and the reproducibility of the CART model, we performed sensitivity analyses using three additional random samples as the training set and compared the results with the main model. We also conducted a sensitivity analysis of the CART approach with a different minimum number of counties (100) in a terminal node.

In contrast to CART which relies on only one tree, random forest creates and aggregates an ensemble of trees using random variable selection and bootstrap sampling^13^. It then takes an average of the outputs of these trees as a prediction. Next, the mean decrease in node impurity is used to calculate variables’ relative importance in predicting the outcome. We created 20,000 trees incorporating all risk factors as predictors. The number of variables randomly sampled as candidates at each tree split was set to 5.

SAS v9.4 was used for data management activities. R v3.6.1 was used for the machine learning analyses (packages “partykit”—ctree for CART and “randomForest” for random forest). Python 3.10.6 (packages “geopandas” and “matplotlib”) was used for maps in Figs. 2, S1. ArcGIS Pro v2.7.0 was used for maps in Fig. 3.

## Results

**Figure 1:**
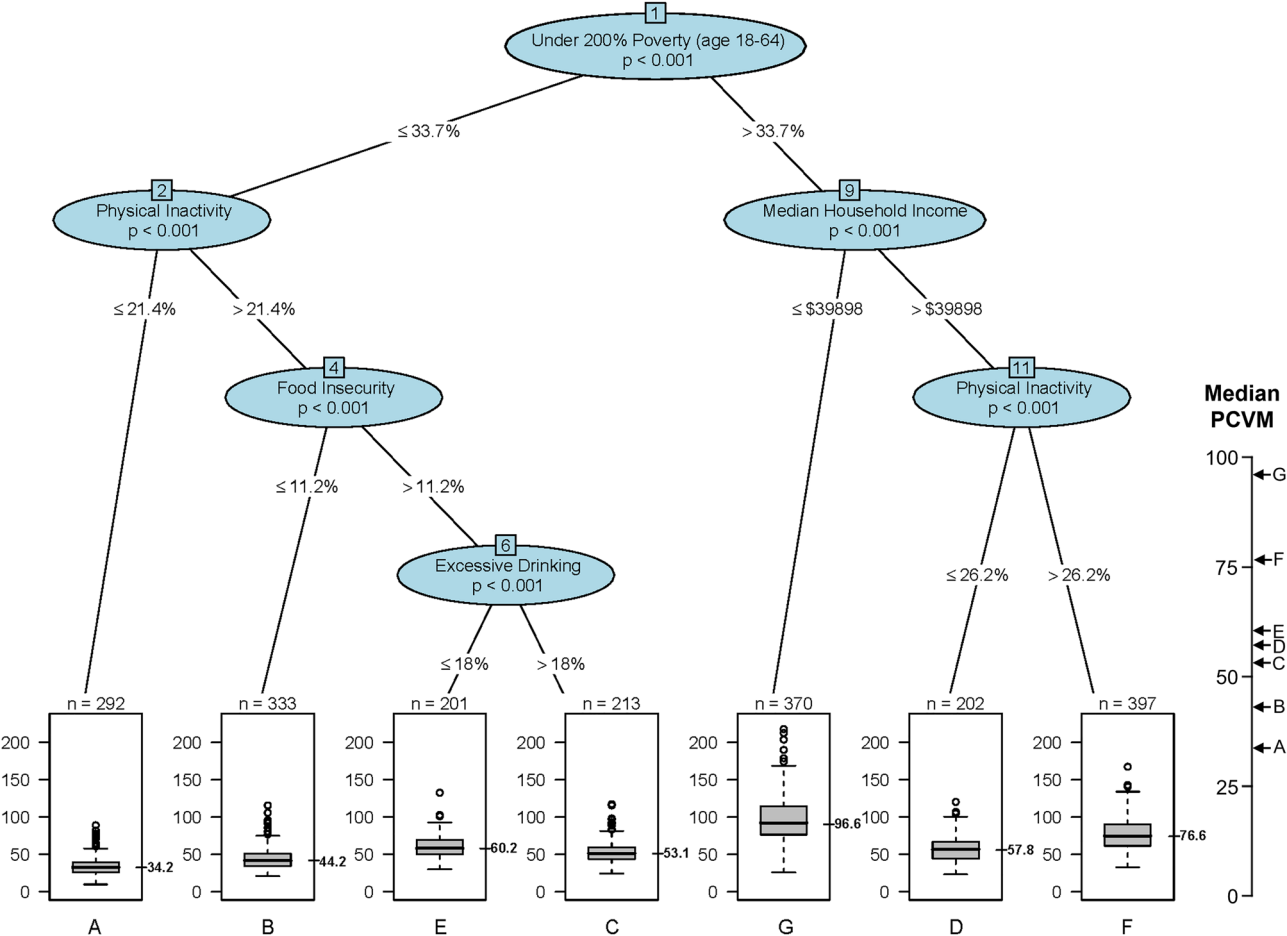
Classification and regression tree analysis (200 minimum counties at a terminal node) to predict county-level premature cardiovascular mortality (PCVM) using counties in the training set (N = 2008). Notes: Each path down to a terminal node represents a county phenotype. Box plots in the terminal nodes represent age-adjusted PCVM (per 100,000 people).

The study included 2509 counties, representing a total of 604,810 deaths from PCVM. There were 2008 and 501 randomly sampled counties in the training set and the test set, respectively. The baseline county characteristics were similar between the training and test sets as shown in Supplemental Table S1. The CART analysis identified seven phenotypes (A to G, in ascending order of the median PCVM) using the training dataset (n = 2008) (Fig. 1). The algorithm selected five variables from all candidate predictors serving as the six splitting nodes in the outcome tree, with *under 200% of poverty* at the top of the tree followed sequentially by *physical inactivity*, *median household income*, *food insecurity*, *physical inactivity*, and *excessive drinking*. All splits were statistically significant (*p* < 0.001).

**Figure 2:**
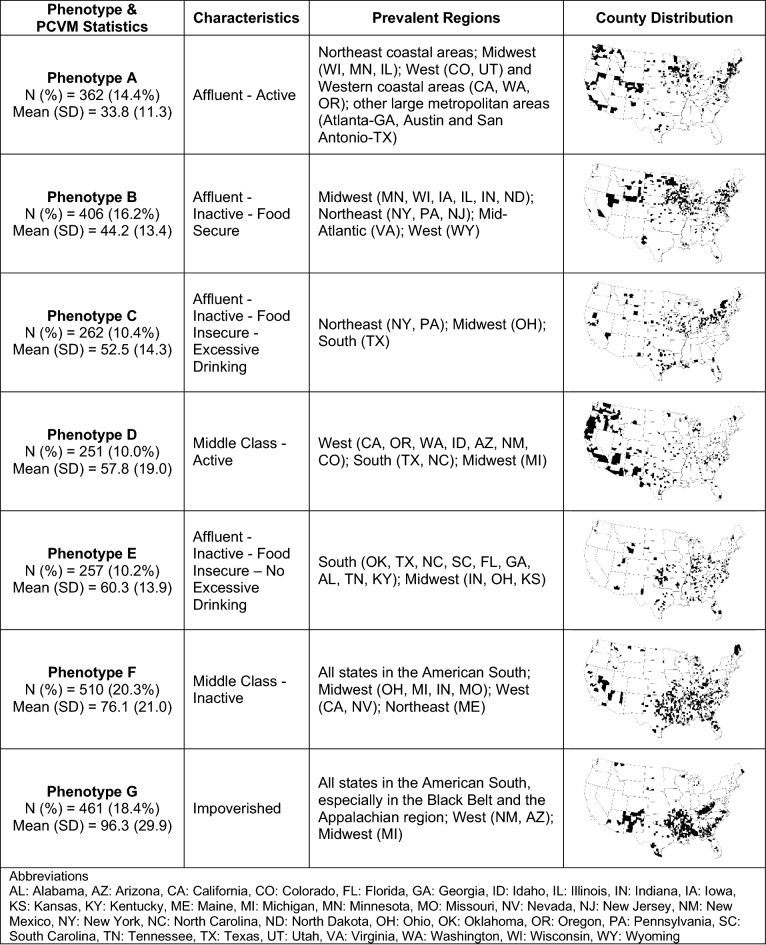
Characteristics of county premature cardiovascular mortality (PCVM) phenotypes identified by CART. Notes: Counties in training and test sets were both included. Maps were created by Python v3.10.6 (https://www.python.org/) and its libraries: geopandas (v0.11.1) and matplotlib (v3.5.3).

Applying the CART model to the test dataset showed no substantial differences in the PCVM distributions versus the training dataset (Supplementary Fig. S2). We summarized the statistics, characteristics, as well as geographic distribution of the identified phenotypes in Fig. 2, including counties in both training and test sets.

On the right side of the tree (Fig. 1), phenotype G (Impoverished) had the highest median PCVM (96.6) among all phenotypes, consisting of counties with more people (aged 18–64) under 200% of the federal poverty level (> 33.7%) and a lower median household income (≤ $39,898). Compared to phenotype G counties, counties of both phenotypes D (Middle Class—Active) and F (Middle Class—Inactive) had a lower median PCVM. Phenotype F counties differentiated from those of Phenotype D by having more people who were physically inactive.

On the left side of the tree (Fig. 1), all counties had fewer people (aged 18–64) under 200% of the federal poverty level and generally had lower rates of PCVM (except for phenotype E counties). Phenotype A (Affluent—Active), with a lower physical inactivity rate (≤ 21.4%), had the lowest median PCVM (34.2), about a third of the median PCVM for phenotype G (96.6). With more people who were physically inactive, phenotypes B (Affluent—Inactive—Food Secure), C (Affluent—Inactive—Food Insecure—Excessive Drinking), and E (Affluent—Inactive—Food Insecure—No Excessive Drinking) also had a higher median PCVM compared to phenotype A. Food insecurity further distinguished phenotype B with C and E, where phenotype B had fewer people who lack adequate access to food (≤ 11.2%) and had about 9 to 16 fewer deaths from CVD per 100,000 people compared to phenotypes C and E. Excessive drinking further separated phenotypes C and E, where phenotype C had more adults reporting binge or heavy drinking and a slightly lower median PCVM compared to phenotype E (53.1 vs. 60.2).

We calculated the county-level PCVM rates of each CVD subtype and grouped counties according to the phenotypes identified by the main model. Supplementary Fig. S3 shows that, for each subtype of PCVM, the median rates of PCVM grouped by phenotype were in ascending order from phenotype A to G, which is consistent with the main model.

The results of the sensitivity analysis of the CART model using three additional samples of counties as the training set were shown in Supplementary Fig. S4. We noticed that the top three nodes (under 200% poverty, physical inactivity, and median household income) in the additional models were the same as in the main model in Fig. 1, despite their splitting values being slightly different. In all four models, physical inactivity was the next splitting node after median household income. Food insecurity and excessive drinking, two variables that were present in the main model, appeared once and not at all, respectively, in the additional models. Poverty, a variable not presented in the main model, was present in all additional models. The results of the sensitivity analysis suggest that CART was relatively stable to changes in data structure, especially for the top splitting variables.

The sensitivity analysis of the CART model with a minimum number of 100 counties in a terminal node included more splitting nodes as well as more phenotypes in the model output (Supplementary Fig. S5), suggesting that additional risk factors were significantly associated with county-level PCVM in different subgroups of the population. These additional splitting variables included *broadband access*, *uninsured (age 18–64)*, *smoking*, and *receipt of Supplemental Nutrition Assistance Program (SNAP) benefits*. Supplementary Fig. S6 illustrates the CART model applied to the test dataset, which revealed no significant differences compared to the model derived from the training dataset.

**Figure 3:**
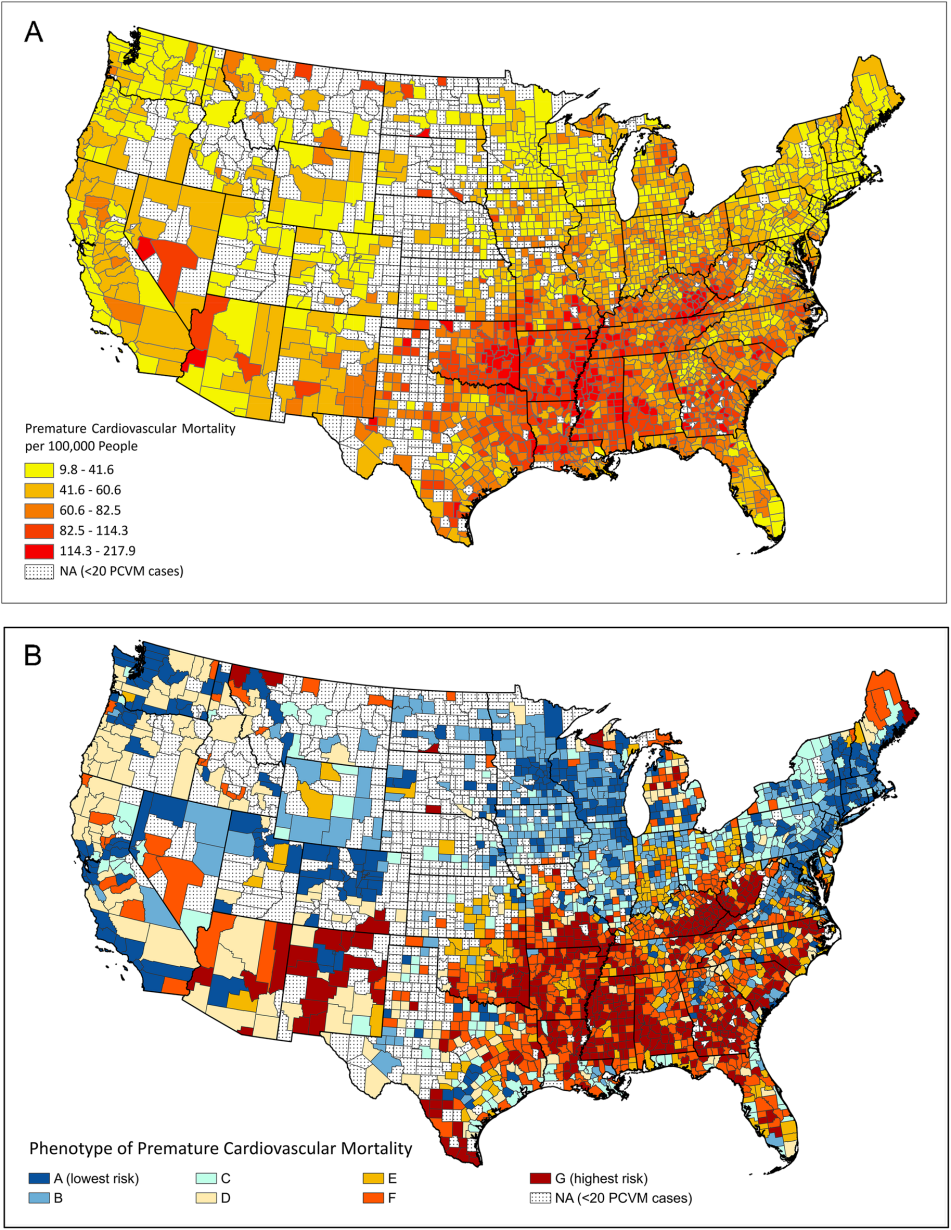
US County Maps of (**A**) age-adjusted premature cardiovascular mortality (per 100,000 people), and (**B**) county phenotypes of premature cardiovascular mortality. Note: maps were created by ArcGIS Pro v2.7.0 (https://pro.arcgis.com/).

Figure 3A,B present the geographic distributions of the county-level PCVM and the phenotypes (for counties in both the training and test sets) from the main model. We observed that counties with high PCVM were mostly in the Southern US. Most of these counties corresponded to the highest-risk phenotypes G (Impoverished) and F (Middle Class—Inactive), which were mostly distributed across the American South and the Appalachian region, especially in Kentucky, West Virginia, Mississippi, Arkansas, southern Alabama, southern Georgia, southern Missouri, and New Mexico for phenotype G. In contrast, many populous coastal counties in the Northeast and the West were of phenotype A (Affluent—Active), the lowest-risk phenotype. Counties of phenotype B (Affluent—Inactive—Food Secure), the second lowest risk phenotype, were mostly found in the Northeast and the Midwest. A large proportion of counties of phenotype C (Affluent—Inactive—Food Insecure—Excessive Drinking) were found in rural New York and Pennsylvania, as well as in many counties in the Midwest, West, and the state of Texas. Many counties of phenotype D (Middle Class—Active), the median-risk phenotype, were in rural areas of the West, including Arizona, California, Oregon, Washington, and Idaho. Counties of phenotype E (Affluent—Inactive—Food Insecure—No Excessive Drinking) were scattered in a few states in the South and the Midwest, such as Oklahoma, Indiana, and North Carolina.

**Figure 4:**
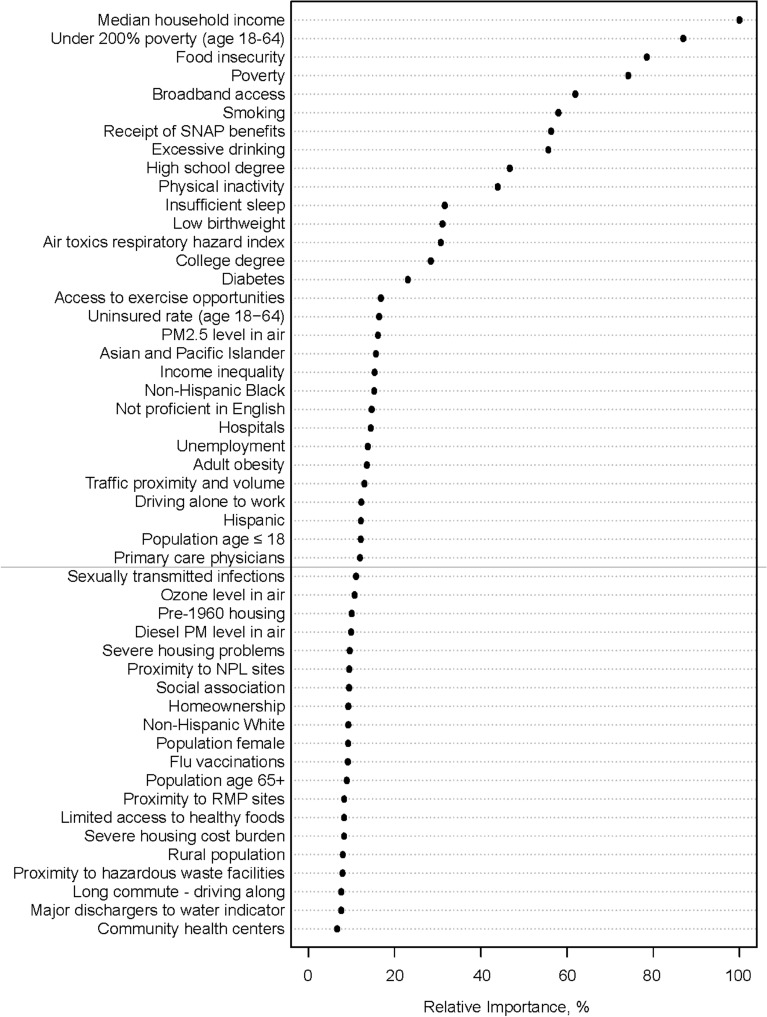
Relative importance plot of risk factors in predicting county-level age-adjusted premature cardiovascular mortality from the random forest analysis. Notes: the most important variable is at the top and scaled to 100%. The importance of the rest of the variables is shown relative to the top variable. Abbreviations: *SNAP* supplemental nutrition assistance program; *PM* fine particulate matter; *RMP* risk management plan; *NLP* national priorities list.

The relative importance of risk factors in predicting PCVM in Fig. 4 suggested that variables that appeared in the CART output were also among the top-ranking variables in the random forest analysis. Notably, median household income, under 200% poverty, and food insecurity were the top three important variables in the random forest plot. Other high-importance variables included broadband access, smoking, and receipt of SNAP benefits, which also appeared in the output of the CART analysis with a minimum number of 100 counties in terminal nodes (Supplementary Fig. S3). Excessive drinking, high school degree, and physical inactivity were ranked 8th to 10th in the variable importance plot.

## Discussion

Our study identified county phenotypes of PCVM and examined their geographic distributions using machine learning approaches and geographic information systems. We found an approximately threefold difference in the PCVM comparing the highest-risk phenotype in the American South, an area termed the stroke belt due to high rates of stroke^16^, with the lowest-risk phenotype in the coastal areas in the Northeast and the West.

Our findings suggest that counties of the highest-PCVM-risk phenotype were highly impoverished. The association between poverty and PCVM has been identified by numerous studies^1–3^. Our study further affirms that income/poverty was the most important predictor of PCVM among various other risk factors related to environmental exposure, health status, health behaviors, and other aspects of socioeconomic status. Previous studies also suggest that physical inactivity was a strong risk factor for PCVM^1–3^. Our study additionally demonstrated that physical inactivity may be more important in predicting PCVM among counties with higher income than those with lower income (as seen that physical inactivity was a splitting node in the lower poverty group or the higher median household income group in Fig. 1). Similarly, food insecurity, an indicator of dietary behavior and socioeconomic status, may have a stronger association with PCVM among counties with higher physical inactivity (i.e., phenotypes A vs. B). These findings suggest that there may be effect measure modifications between risk factors and their association with PCVM, as may be the case between poverty and physical inactivity, or between physical inactivity and food insecurity.

Notably, counties of the impoverished phenotype (G) and the Middle Class—Inactive phenotype (F), the two highest-risk phenotypes, were mostly located in the American South and the Appalachian region. The concentration of these two phenotypes in the same geographic area provides an opportunity to study in greater detail the interaction between poverty and physical inactivity in the causal pathway to PCVM.

Our study included multiple environmental risk factors in the models. Environmental exposures, especially air pollution, have been mechanistically and epidemiologically linked with disproportionate cardiometabolic outcomes^17–19^. However, none of the environmental factors appeared in the CART output, nor were they listed as the top ten variables in the random forest plot. On the other hand, multiple studies have demonstrated remarkable overlap between several environmental exposures and socioeconomic factors^20^, with significant effect interactions between factors such as air pollution and social vulnerability^7^. One reason behind this discordance is that individuals within counties may have been disproportionately exposed to pollutants, and it is difficult to evaluate to which groups and to what extent of individuals were exposed to the pollutants using data from the current study. Future studies should focus on associations between environmental factors and PCVM at a finer geographic scale.

We also note that risk factors not presented in the CART output may be still highly associated with PCVM, such as broadband access, smoking, receipt of SNAP benefits, and high school education, as suggested by the random forest variable importance plot.

There are several methodological advantages that lend confidence to our study. First, unlike traditional statistical methods (such as regression analysis), CART and random forest machine learning methods can handle a large number of highly correlated variables simultaneously without concerns about multicollinearity due to their variable selection and bootstrap sampling strategies. A second advantage of our methods is that CART has the advantage of visualizing and conceptualizing phenotypes, while random forest complements CART in risk factor importance evaluation and model stability. Specifically, CART selects variables and presents “pathways” for each observation towards its “destination”, where the characteristics along the “pathways” can be used to determine phenotypes associated with PCVM. On the other hand, random forest evaluates all risk factors on their relative importance, including those not selected by CART. Additionally, the variable importance plot of random forest is less sensitive to changes in the data (such as using different years of data) compared to the result of the single-tree CART algorithm.

The above advantages of using CART and random forest methods, together with geographic information systems, have been demonstrated in a prior study investigating the phenotypes of late-stage breast cancer diagnosis^21^ and cancer mortality^22^. This study further demonstrates the validity of this approach in uncovering the combination of risk factors and their relative importance in predicting county-level PCVM.

### Limitations

The findings of our study should be interpreted within the context of its limitations. First, the accuracy of diagnostic codes from death certificates cannot be ascertained, and there might be additional exposures and proximal contributors to mortality that we were not able to capture. Second, the data collection period for the risk factors did not perfectly match that for the PCVM data, which may be problematic if there is a temporal lag in the effect of risk factors on PCVM. Future studies should explore temporal associations between risk factors and PCVM. Third, data for many risk factors were collected from self-reported surveys based on a sample of the population, where the quality of reporting, response rates, and selection bias may impact the accuracy of the measures. Fourth, to ensure statistical stability, our analyses excluded counties with less than 20 deaths caused by CVD, which might have led to a bias towards less populated areas, especially in the many states in the West and Midwest. Future studies should consider regionalization methods, such as the Max-P-regions model^23^, to combine counties with small numbers of cases. Finally, counties are relatively large geographic units with seemingly heterogeneous populations and exposures. Whether the associations discovered in the current study are also present in smaller geographic scales (e.g., census tracts or block groups) or at the individual level with long-term cardiovascular outcomes remains to be elucidated. Nevertheless, this proof-of-concept study provides a platform for characterizing the relationships between community-level risk factors and health outcomes.

## Conclusion

The use of CART and random forest machine learning methods and geographic information systems can help uncover risk factor associations in predicting PCVM. Interventions to reduce PCVM should be tailored and target geographic areas with high-risk phenotypes of PCVM.

## Supplementary Information

Supplementary Information.

## Data Availability

The data sets generated during this study are available from the corresponding author upon reasonable request.
