# Author Correction: Risk factors and geographic disparities in premature cardiovascular mortality in US counties: a machine learning approach

**DOI:** 10.1038/s41598-023-32047-z

**Published:** 2023-03-27

**Authors:** Weichuan Dong, Issam Motairek, Khurram Nasir, Zhuo Chen, Uriel Kim, Yassin Khalifa, Darcy Freedman, Stephanie Griggs, Sanjay Rajagopalan, Sadeer G. Al‑Kindi

**Affiliations:** 1 Department of Population and Quantitative Health Sciences, Case Western Reserve University School of Medicine, Cleveland, OH 44106 USA; 2 Harrington Heart and Vascular Institute, University Hospitals, 11100 Euclid Ave, Cleveland, OH 44106 USA; 3 Houston Methodist Hospital, Houston, TX 77030 USA; 4 Kellogg School of Management, Northwestern University, Evanston, IL 60208 USA; 5 Mary Ann Swetland Center for Environmental Health, Case Western Reserve University, Cleveland, OH 44106 USA; 6 Frances Bolton School of Nursing, Case Western Reserve University, Cleveland, OH 44106 USA; 7 Case Western Reserve University School of Medicine, Cleveland, OH 44106 USA

Correction to: *Scientific Reports* 10.1038/s41598-023-30188-9, published online 20 February 2023

The original version of this Article contained an error in the Results section.

“Phenotype F counties differentiated from those of Phenotype D by having a 33% higher percentage of people who were physically inactive.”

 now reads:

“Phenotype F counties differentiated from those of Phenotype D by having more people who were physically inactive.”

The original Article has been corrected.

