# Supplementary material for: Risk factors and geographic disparities in premature cardiovascular mortality in US counties: a machine learning approach
Source: Sci Rep. 2023 Feb 20;13:2978. doi: 10.1038/s41598-023-30188-9 (PMC9941082; doi:10.1038/s41598-023-30188-9)

**Supplementary Information**

Risk Factors and Geographic Disparities in Premature Cardiovascular Mortality in US Counties: A Machine Learning Approach

**Supplemental Table S1.** Baseline county characteristics between the training and test sets. Note: p values were based on t-tests.

| **Variables** | **Training (n=2008)** | **Testing (501)** | **p value** |
| --- | --- | --- | --- |
| **Race/ethnicity** | | | |
| Hispanic | 9.0 (13.2) | 9.8 (13.6) | 0.271 |
| Non-Hispanic Black | 10.7 (15.1) | 10.5 (15.1) | 0.777 |
| Non-Hispanic White | 75.5 (19.8) | 75.0 (19.8) | 0.644 |
| Asian and Pacific Islander | 1.7 (2.7) | 1.8 (2.9) | 0.644 |
| **Population structure** | | | |
| Population age ≤ 18 | 22.2 (3.1) | 22.1 (3.2) | 0.544 |
| Population age 65+ | 18.1 (4.1) | 18.5 (4.9) | 0.115 |
| Population female | 50.2 (2) | 50.2 (1.9) | 0.765 |
| Rural population | 52.8 (29.3) | 51.1 (30.1) | 0.262 |
| **Environmental exposure** | | | |
| PM2.5 level in air | 8.0 (1.2) | 8.1 (1.2) | 0.291 |
| Air toxics respiratory hazard index | 0.38 (0.13) | 0.39 (0.14) | 0.406 |
| Ozone level in air | 41.5 (4.9) | 41.3 (4.9) | 0.323 |
| Diesel PM level in air | 0.23 (0.16) | 0.24 (0.18) | 0.359 |
| Traffic proximity and volume | 178.8 (317) | 191 (322.2) | 0.446 |
| Pre-1960 housing (lead paint indicator) | 0.27 (0.14) | 0.26 (0.14) | 0.303 |
| Proximity to RMP sites | 0.48 (0.47) | 0.51 (0.52) | 0.277 |
| Proximity to hazardous waste facilities | 0.76 (3.13) | 1.18 (9.88) | 0.352 |
| Proximity to NPL sites | 0.07 (0.10) | 0.07 (0.10) | 0.862 |
| Major dischargers to water indicator | 18.4 (693.6) | 7.3 (104.4) | 0.493 |
| **Socioeconomic status** | | | |
| Income inequality | 4.6 (0.8) | 4.6 (0.7) | 0.765 |
| High school degree | 86.6 (5.9) | 86.5 (5.8) | 0.753 |
| College degree | 57.5 (11.4) | 57.6 (11.3) | 0.786 |
| Unemployment | 4.7 (1.5) | 4.8 (1.6) | 0.476 |
| Median Household Income | 51245 (14021) | 51174 (14068) | 0.939 |
| Poverty | 15.8 (6.3) | 15.8 (6.0) | 0.973 |
| Income < 200% of federal poverty level (age 18-64) | 33.3 (9.3) | 33.6 (9.1) | 0.550 |
| Receipt of SNAP benefits | 14.3 (6.8) | 14.4 (6.7) | 0.937 |
| not proficient in English | 1.6 (2.6) | 1.8 (2.6) | 0.231 |
| Severe housing problems | 14.0 (3.8) | 14.1 (3.6) | 0.411 |
| Severe housing cost burden | 11.4 (3.3) | 11.5 (3.3) | 0.373 |
| Homeownership | 71.2 (8.2) | 70.7 (8.0) | 0.299 |
| Broadband access | 75.7 (8.9) | 75.8 (9.0) | 0.813 |
| Social associations | 4.1 (0.7) | 4.1 (0.7) | 0.108 |
| **Health status** | | | |
| Diabetes | 12.7 (3.8) | 12.6 (3.5) | 0.478 |
| Low birthweight | 8.4 (1.9) | 8.4 (2.0) | 0.942 |
| Sexually transmitted infections | 419.5 (248.9) | 417.4 (236.8) | 0.864 |
| **Health behaviors** | | | |
| Adult obesity | 34.0 (5.8) | 34.0 (5.9) | 0.784 |
| Insufficient sleep | 37.5 (3.8) | 37.4 (3.8) | 0.662 |
| Excessive drinking | 17.4 (3.2) | 17.3 (3.2) | 0.656 |
| Adult smoking | 18.4 (4.7) | 18.6 (4.6) | 0.667 |
| Physical inactivity | 27.1 (6.0) | 27.1 (6.1) | 0.935 |
| Flu vaccinations (Medicare enrollees) | 43.6 (8.4) | 43.1 (9.0) | 0.242 |
| Food insecurity | 13.6 (4) | 13.7 (3.9) | 0.465 |
| Limited access to healthy foods | 7.2 (5.6) | 7.0 (4.8) | 0.434 |
| Access to exercise opportunities | 64.6 (21.8) | 66.0 (22.0) | 0.192 |
| Driving alone to work | 81.1 (5.7) | 80.9 (6.1) | 0.575 |
| Long commute-driving alone | 33.6 (12.1) | 33.2 (12.2) | 0.581 |
| **Clinical Care** | | | |
| uninsured rate (age 18-64) | 13.2 (6.0) | 13.3 (6.1) | 0.929 |
| Primary care physicians | 53.5 (33.3) | 54.8 (31.6) | 0.402 |
| Hospitals | 3.5 (3.5) | 3.5 (3.3) | 0.965 |
| Community health centers | 5.3 (8.5) | 6.1 (11.1) | 0.117 |

**Supplemental Figure S1.** Geographic distributions of county-level risk factors of premature cardiovascular mortality. Note: Maps were created by Python v3.10.6 (https://www.python.org/) and its libraries: geopandas (v0.11.1) and matplotlib (v3.5.3).

| 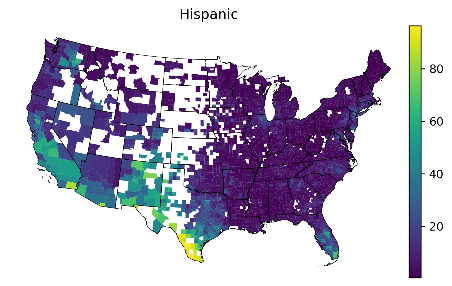 | 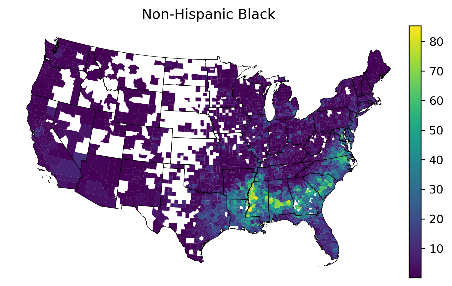 | 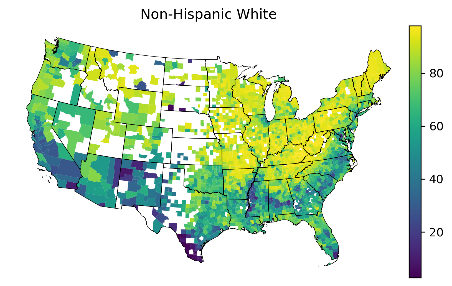 |
| --- | --- | --- |
| 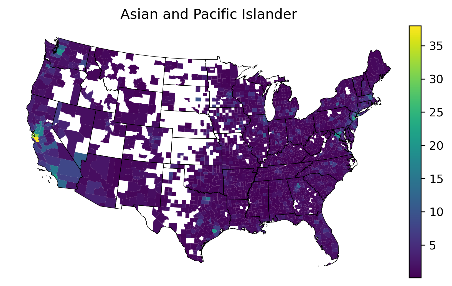 | 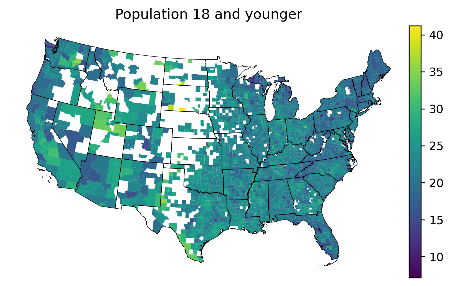 | 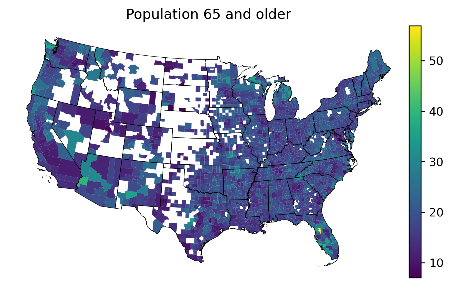 |
| 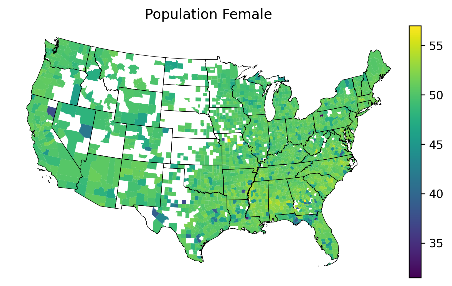 | 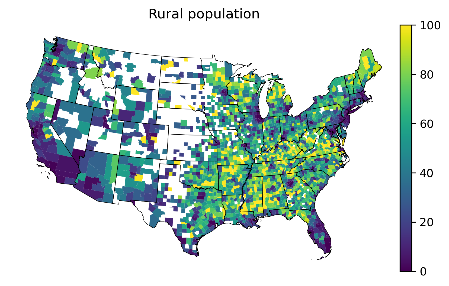 | 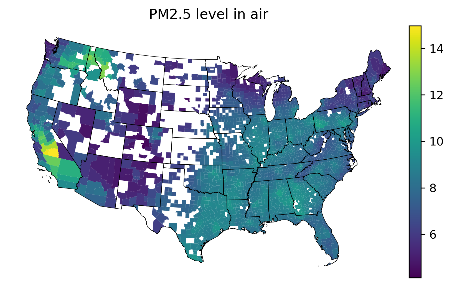 |
| 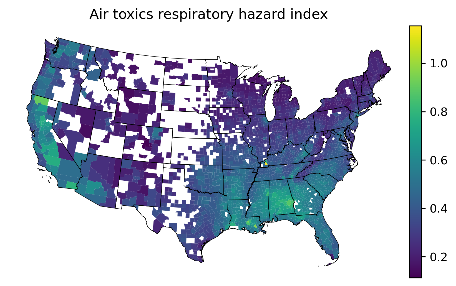 | 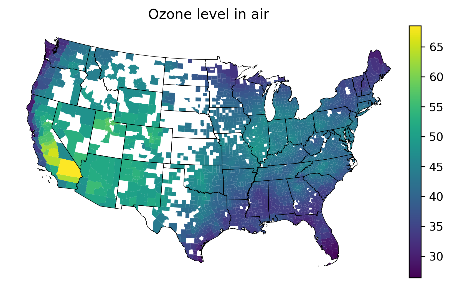 | 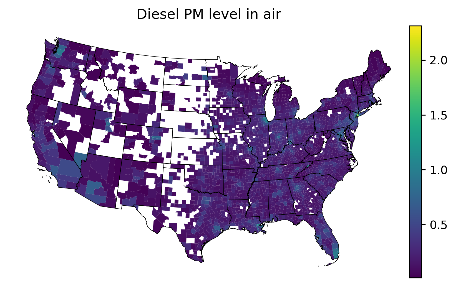 |
| 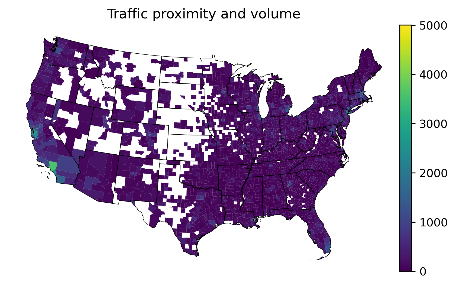 | 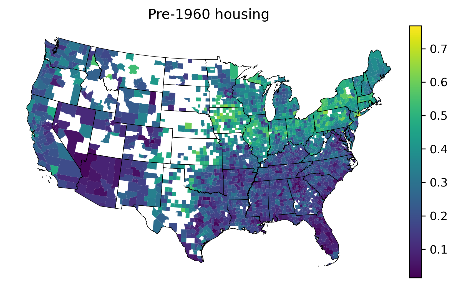 | 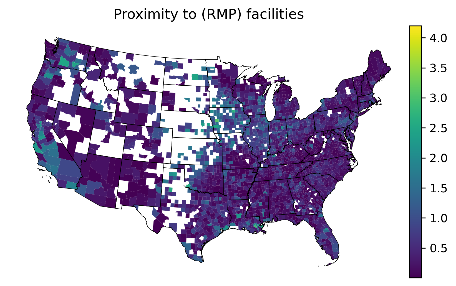 |
| 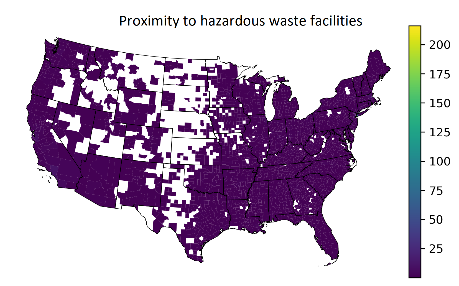 | 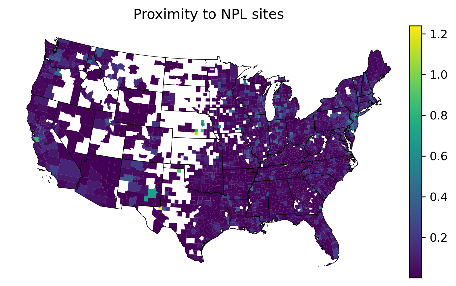 | 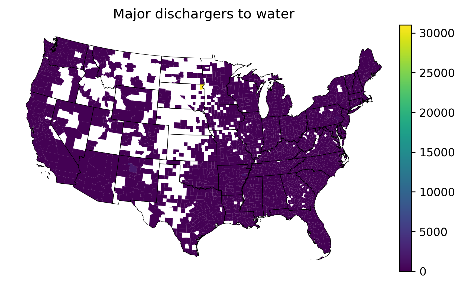 |
| 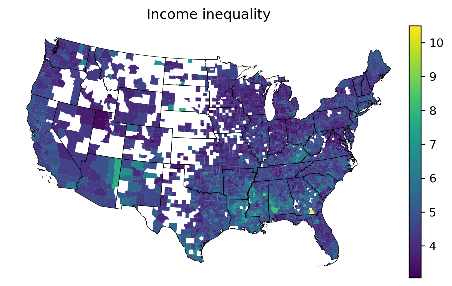 | 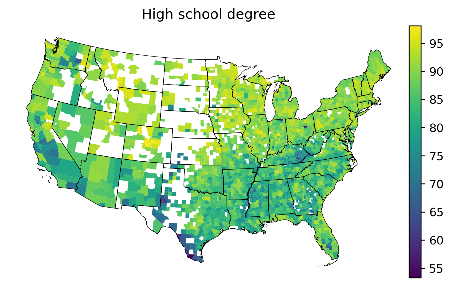 | 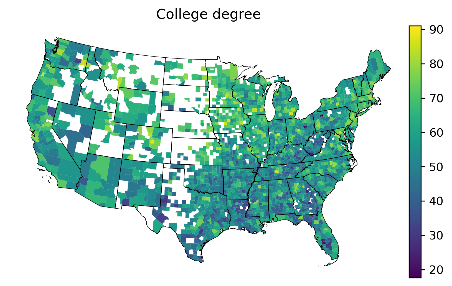 |
| 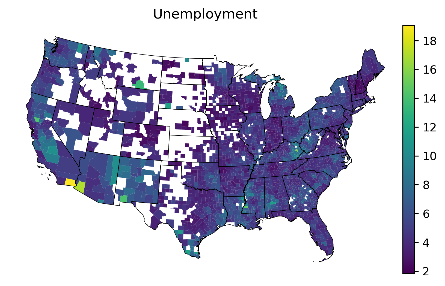 | 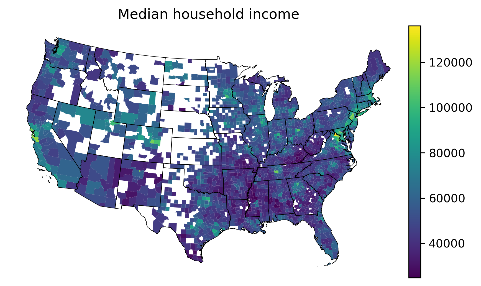 | 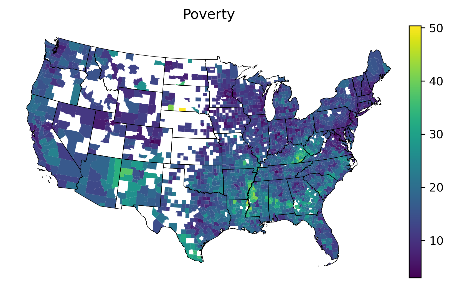 |
| 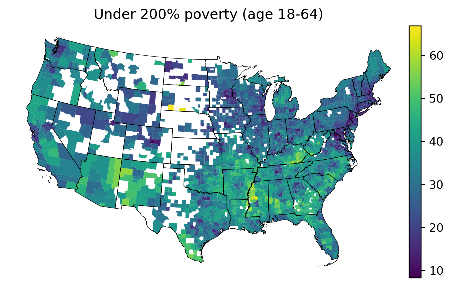 | 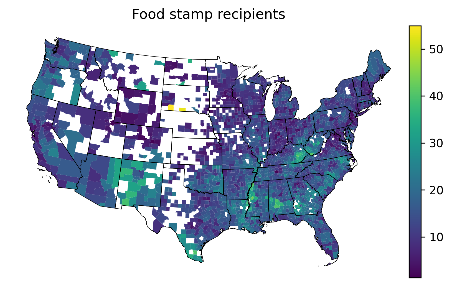 | 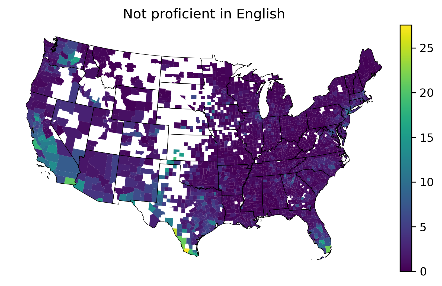 |
| 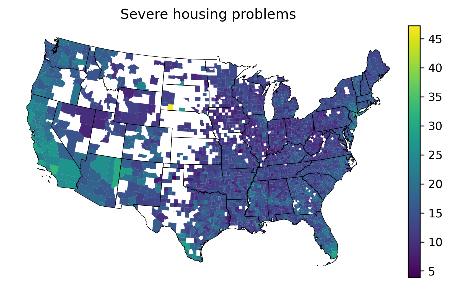 | 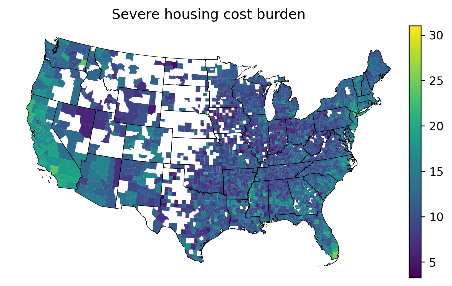 | 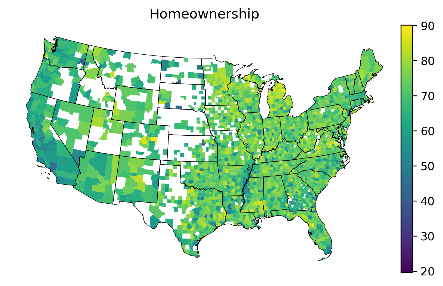 |
| 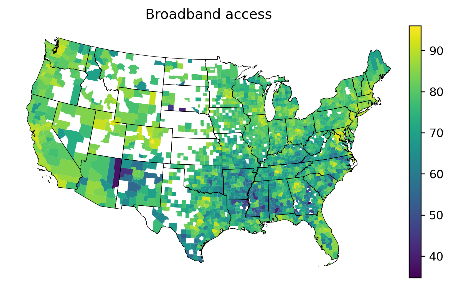 | 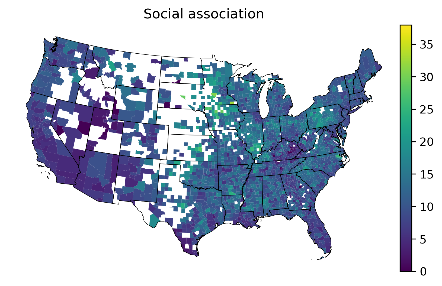 | 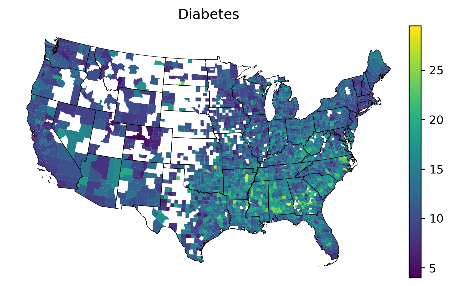 |
| 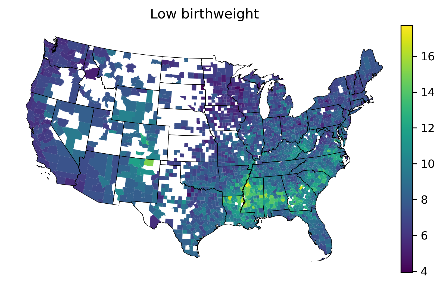 | 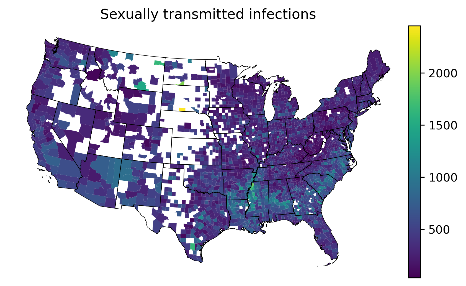 | 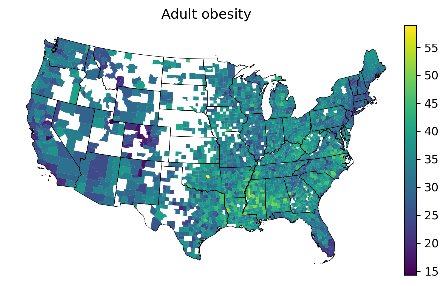 |
| 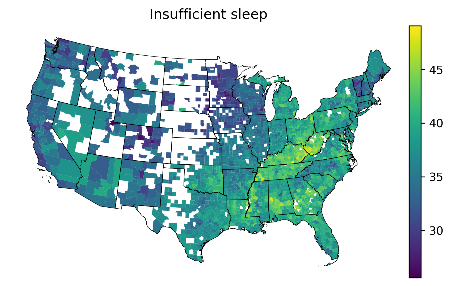 | 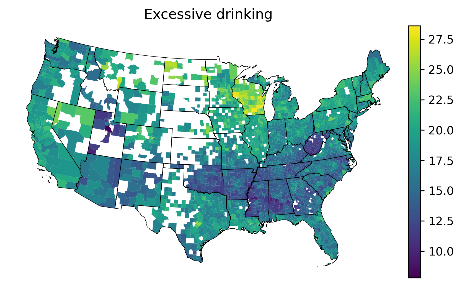 | 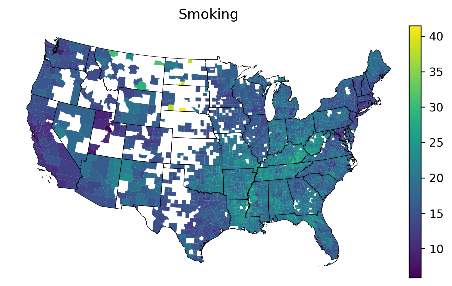 |
| 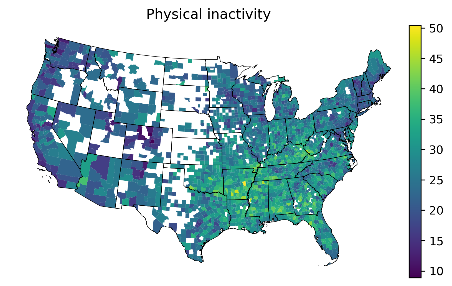 | 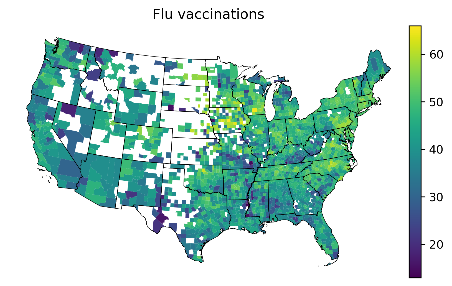 | 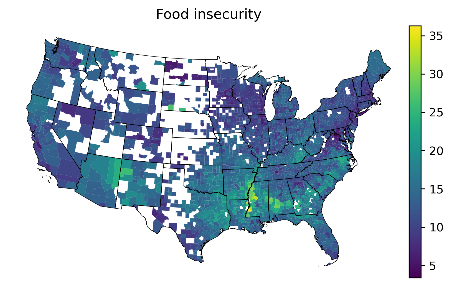 |
| 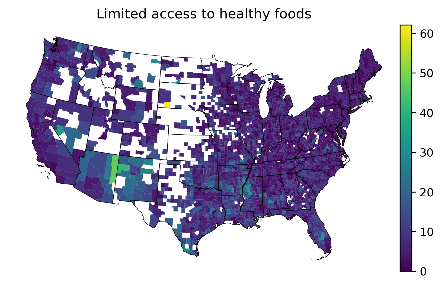 | 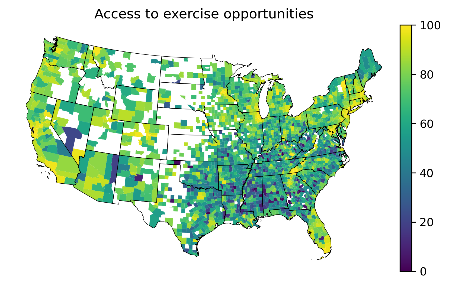 | 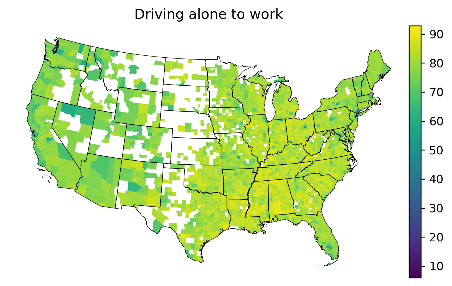 |
| 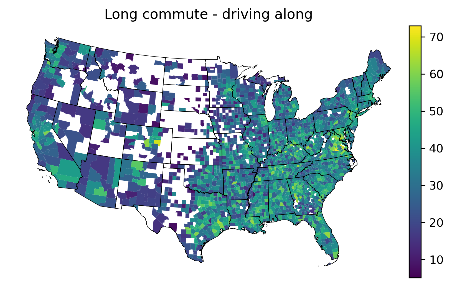 | 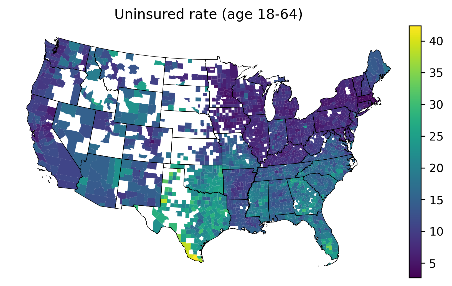 | 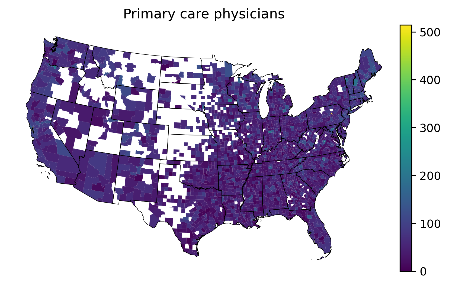 |
| 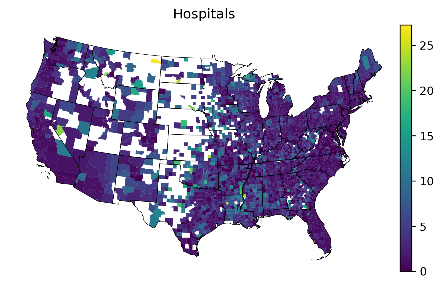 | 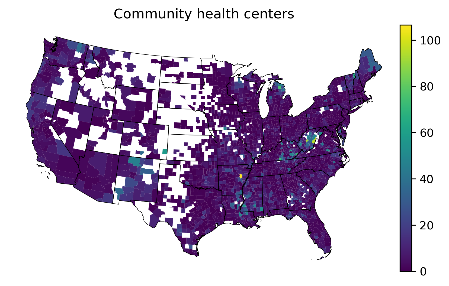 |  |

**Supplementary Figure S2**. Comparisons of premature cardiovascular mortality rates between counties from the training (n=2008) and test (n=501) datasets by county phenotype identified by CART with 200 minimum counties at a terminal node.


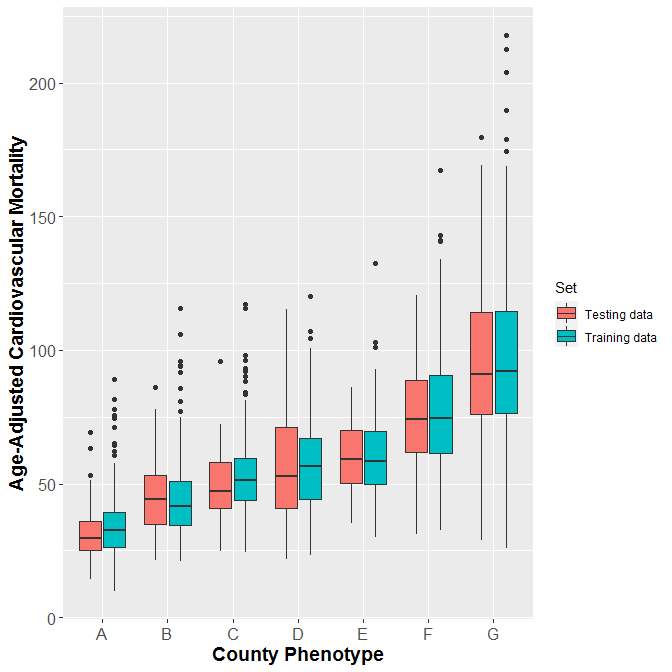


**Supplementary Figure S3**. Comparisons of subtypes of premature cardiovascular mortality rates between counties from the training (n=2008) and test (n=501) datasets by county phenotype identified by CART with 200 minimum counties at a terminal node.


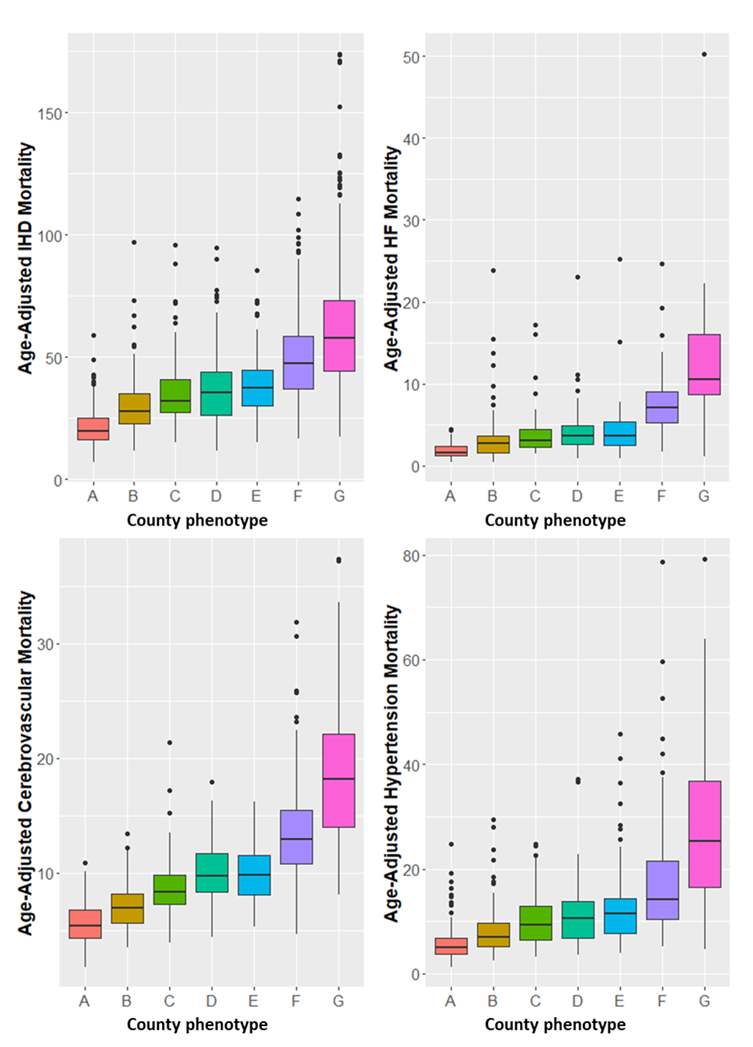


**Supplementary Figure S4**. Classification and regression tree analysis (200 minimum counties at a terminal node) to predict county-level premature cardiovascular mortality (PCVM) using three additional random samples (A, B, and C) as the training set (N=2008). Notes: Each path down to a terminal node represents a county phenotype. Box plots in the terminal nodes represent age-adjusted PCVM (per 100,000 people).

A. Random Sample 1

**
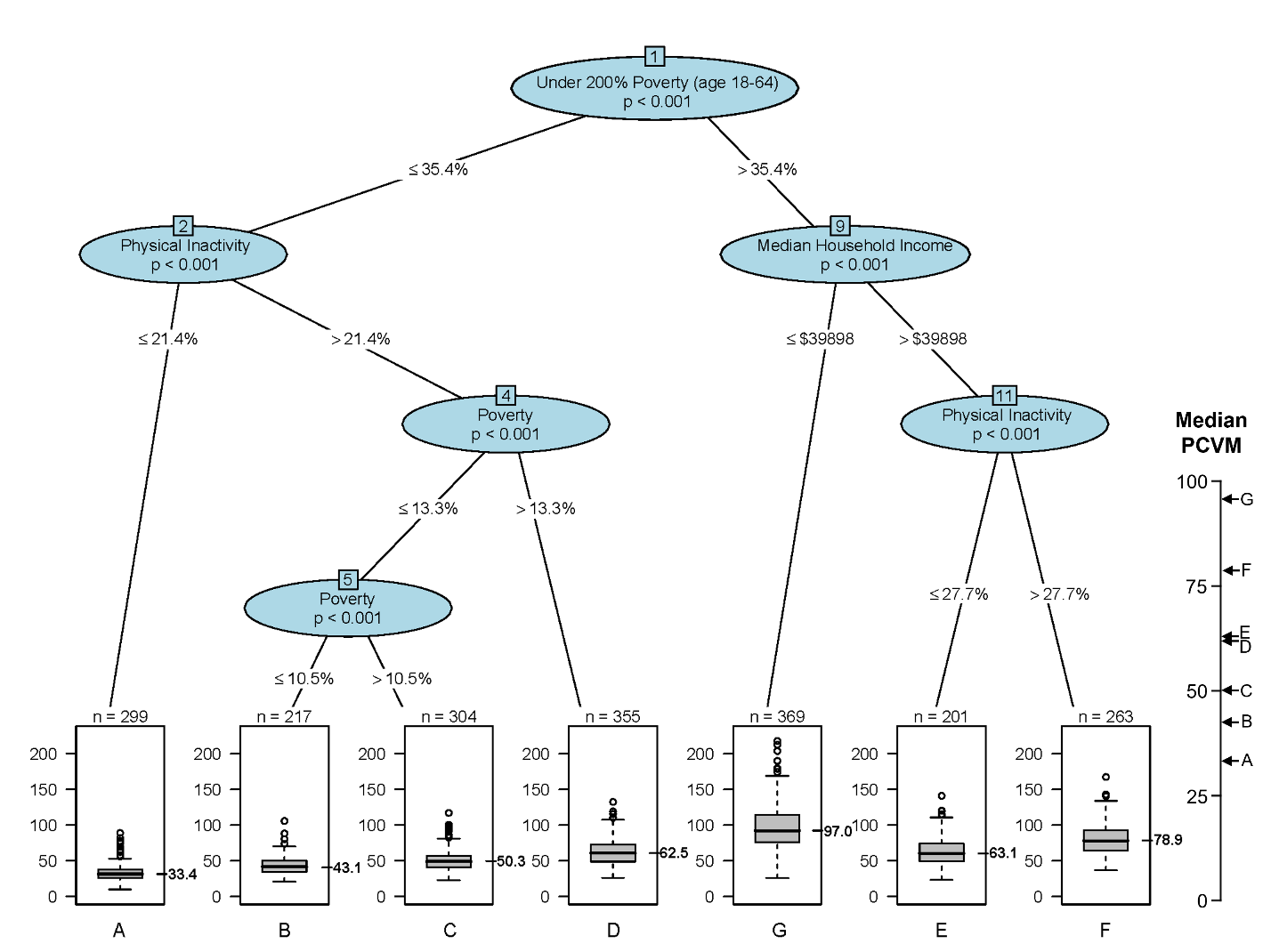
**

B. Random Sample 2**
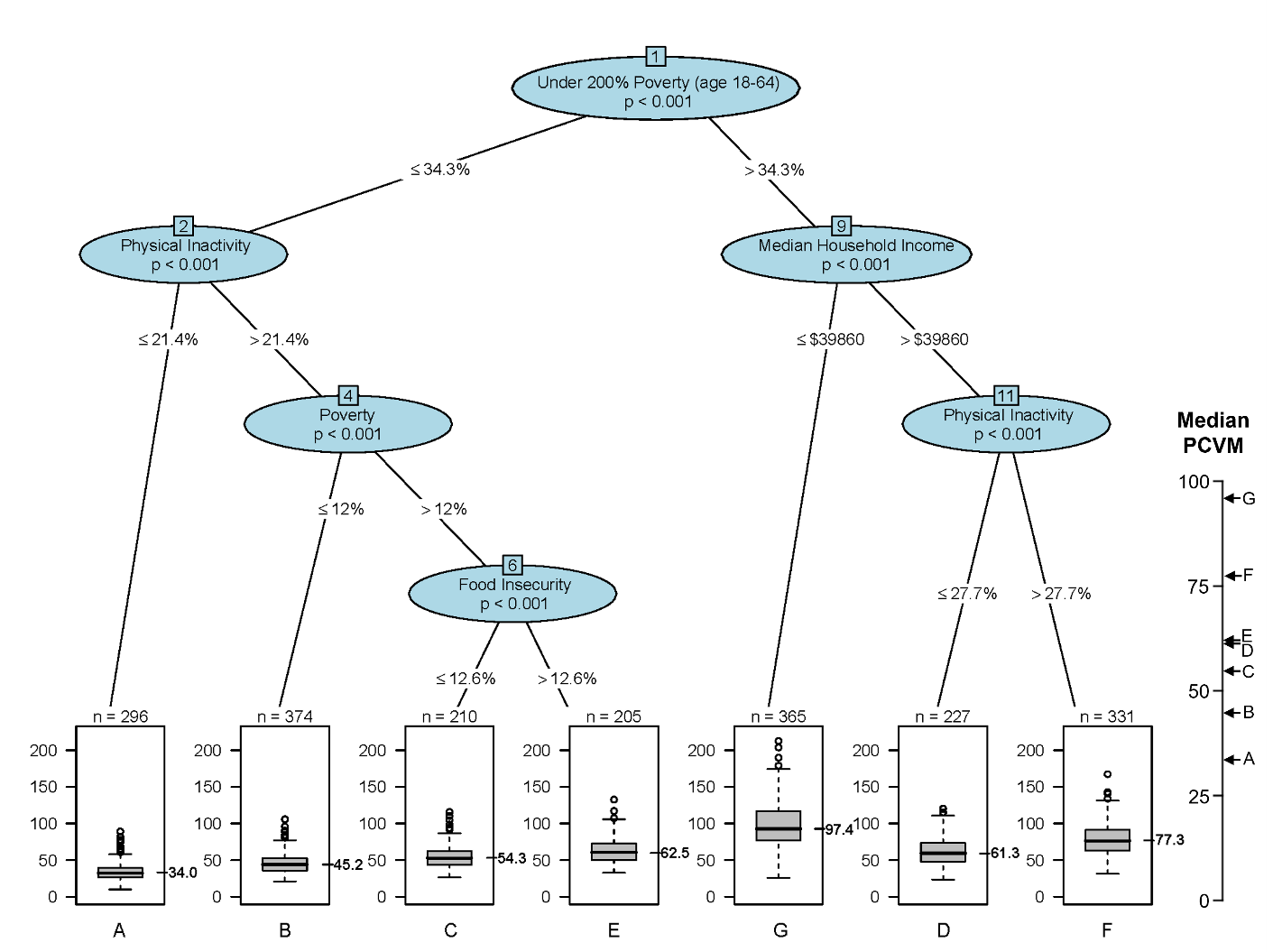
**

C. Random Sample 3

**
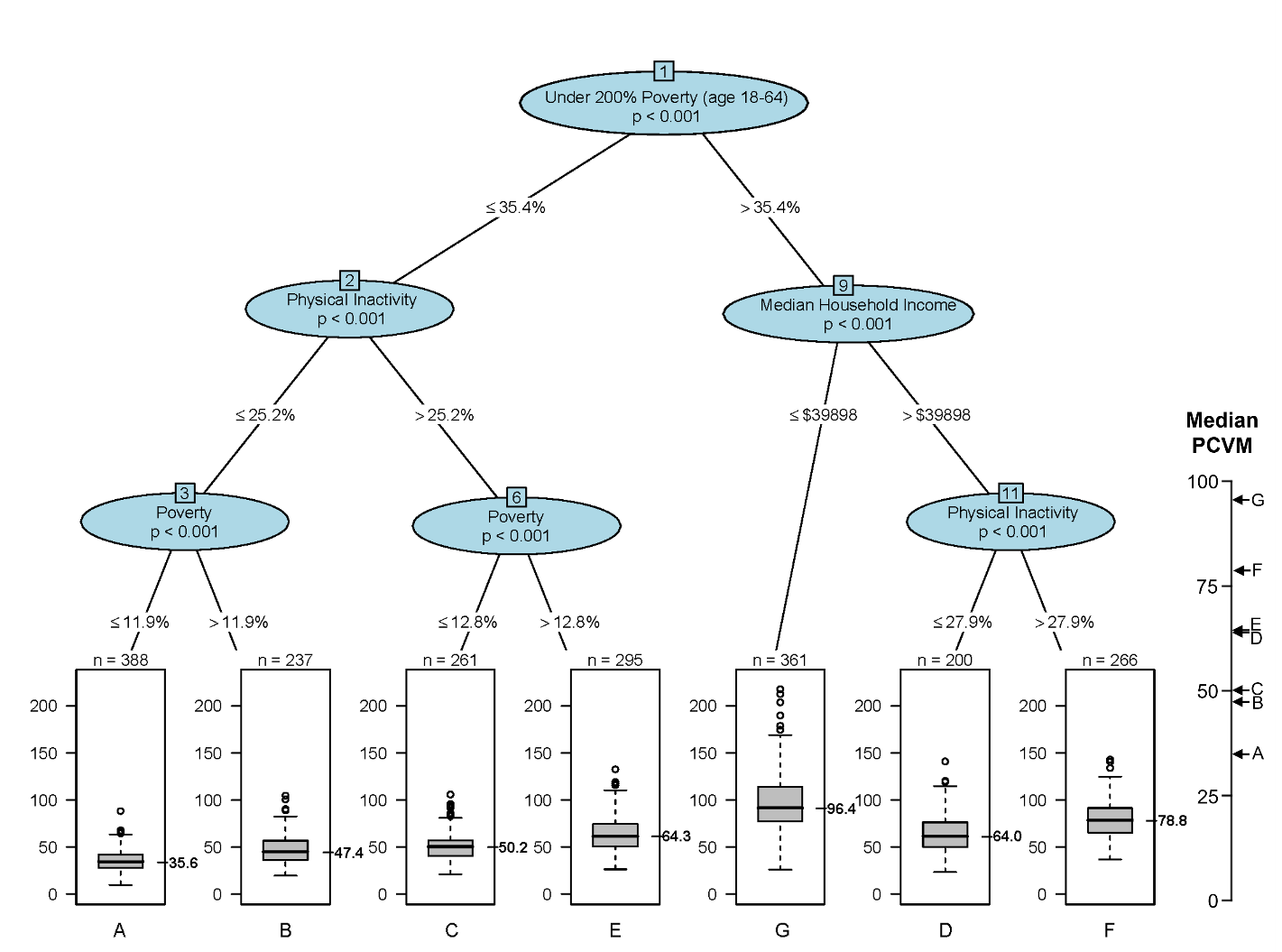
**

**Supplementary Figure S5.** Classification and regression tree analysis (100 minimum counties at a terminal node) to predict county-level premature cardiovascular mortality (PCVM) using counties in the training set (N=2008). Notes: Each path down to a terminal node represents a county phenotype. Box plots in the terminal nodes represent age-adjusted PCVM (per 100,000 people).


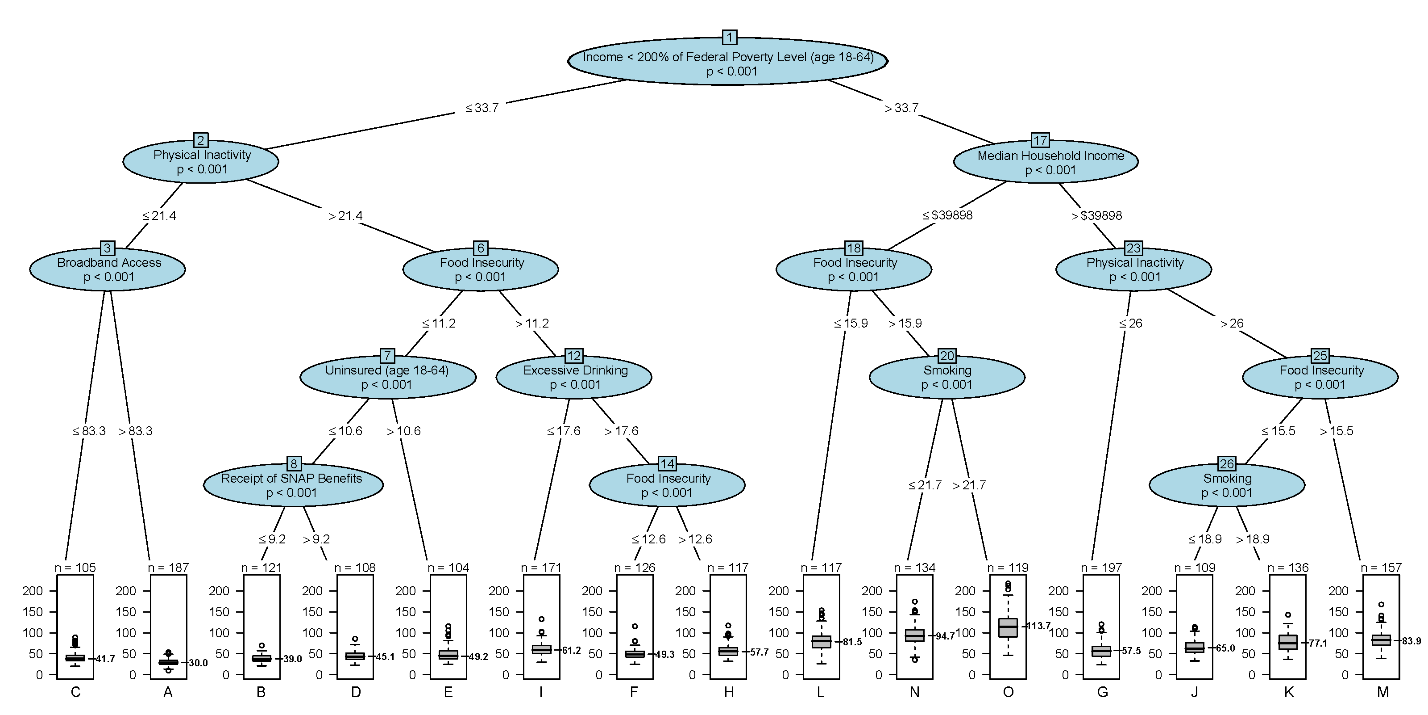


**Supplementary Figure S6**. Comparisons of premature cardiovascular mortality rates between counties from the training (n=2008) and test (n=501) datasets by county phenotype identified by CART with 100 minimum counties at a terminal node.


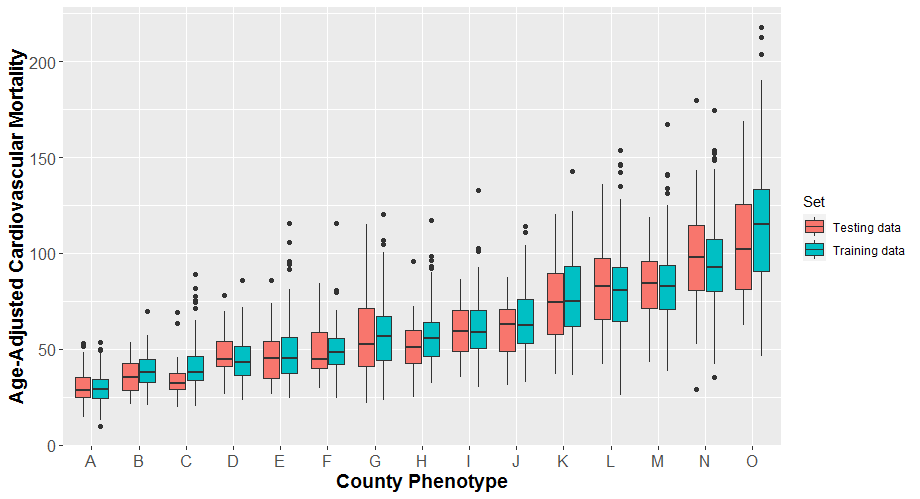

Supplement: Supplementary file 1 — Supplementary Information. [file 41598_2023_30188_MOESM1_ESM.docx]
